# The Netherlands Sports Cardiology Map: a step towards sports cardiology network medicine for patient and athlete care

**DOI:** 10.1007/s12471-020-01530-x

**Published:** 2020-12-23

**Authors:** J. C. van Hattum, S. M. Verwijs, R. Rienks, N. R. Bijsterveld, S. T. de Vries, Y. M. Pinto, A. A. M. Wilde, H. T. Jørstad

**Affiliations:** 1 Department of Cardiology, Heart Center, Amsterdam UMC, University of Amsterdam, Amsterdam Cardiovascular Sciences, Amsterdam, The Netherlands; 2 CardioExpert, Amsterdam, The Netherlands; 3 Department of Cardiology, Flevo Hospital, Almere, The Netherlands; 4 Department of Cardiology, Tjongerschans Hospital, Heerenveen, The Netherlands

**Keywords:** Sports cardiology, Map, Overview, Network, Infrastructure

## Abstract

Sports cardiology is a rapidly evolving subspecialty of cardiology, with a growing demand for expertise. To improve patient care, clinicians, patients, and athletes (recreational to elite) should be able to easily identify specialised care pathways, expertise centres and clinicians with sports cardiology expertise. To this purpose, several international societies and organisations recommend establishing a local and national sports cardiology infrastructure. We therefore aimed to establish The Netherlands Sports Cardiology Map. We conducted a web-based survey, which was published on the Netherlands Society of Cardiology home page (2019–2020) and in which each cardiology department or clinic was asked to provide information on sports cardiology expertise and the current infrastructure. Of the 46 respondent centres, 28 (61%) reported that they had expertise in sports cardiology, of which 22 (79%) had specific expertise in one or more specific types of sports. Integrated multidisciplinary meetings were reported by 43% of the centres (*n* = 12/28). Only two centres reported ongoing research projects that had been approved by an institutional review board. The Netherlands Sports Cardiology Map is an important step towards improving the existing infrastructure and developing network medicine for sports cardiology.

Sports cardiology is a rapidly evolving subspecialty of cardiology, with a growing demand for expertise. According to the Dutch Olympic Committee*Dutch Sports Federation (NOC*NSF), 65% of the Dutch aged 5–80 years participate in sports at least four times per month. In addition, there are 5 million athletes who are associated with sports federations and 800 elite athletes who are registered as (potential) Olympic games participants.

The challenge for sports cardiology healthcare professionals is to maximise safety in sports, through cardiovascular screenings, differentiating cardiac remodelling from pathology, and clinical evaluation and management of symptomatic athletes or athletes with cardiovascular disease, ranging from amateur to elite levels. To this purpose, the American College of Cardiology [1] and the European Society of Cardiology (ESC)[2] have both published (proposed) sports cardiology core curriculums; they also update consensus documents on a regular basis. Sports medicine healthcare professionals have further emphasised the need for multidisciplinary networks, with the American Medical Society for Sports Medicine recommending the establishment of a dedicated infrastructure for local collaborations and partnerships between sports physicians and sports cardiologists. This American society also recommends that regional expert centres are established to assist in electrocardiogram interpretation and evaluation of athletes with suspected or known cardiovascular disorders [3]. In line with this, the Sports Cardiology Section of the European Association of Preventive Cardiology has stated that offering uniform protocolised care for risk stratification and management is an important challenge in the management of athletes with cardiac disease [4–9]. In addition to the establishment of regional expert centres, this requires national and international educational events to update and improve sports cardiologists’ skills in and knowledge of the continuously developing field of athlete care [2].

To address these challenges, the Sports Cardiology Section of the Netherlands Society of Cardiology (*NVVC*) was founded, which aims to promote education, to contribute to national protocols and consensus statements, and to stimulate research in the broad field of sports cardiology. As a first step in establishing further infrastructure and expert centres, regional centres and expertise need to be identified. We therefore aimed to establish The Netherlands Sports Cardiology Map to enable clinicians, patients, and athletes (recreational to elite) to easily identify expertise centres and clinicians with sports cardiology expertise.

## Survey

We conducted a web-based survey, which was published on the NVVC home page (2019–2020). In this survey, each cardiology department or clinic was asked to provide information on sports cardiology expertise and the current infrastructure. We collected data on specific fields of expertise, specific fields of interest and expertise in particular sports. Also, data on the presence of (formalised) collaborations with professional sports clubs or federations, organised (multidisciplinary) sports cardiology meetings or panels (with details of participating disciplines), and ongoing studies approved by an institutional review board (IRB) were collected. Finally, we collected data on (formalised) collaborations with sports physicians, sports federation physicians and NOC*NSF. All data were self-reported; no quality criteria were applied when listing centres of expertise. Based on the collected data, we designed a map with centres of expertise and a list of specific features per centre.

**Fig. 1:**
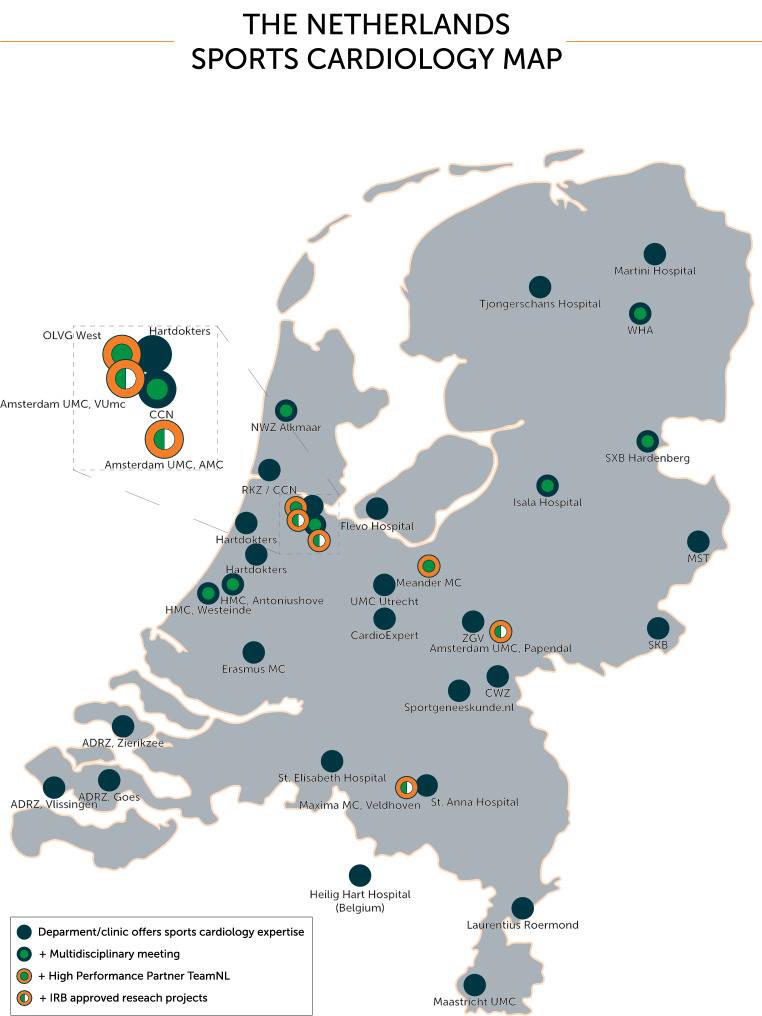
The Netherlands Sports Cardiology Map, consisting of 28 centres with expertise in sports cardiology (1 located in Belgium). Integrated multidisciplinary meetings are held at Amsterdam University Medical Centre (UMC), Cardiology Centre the Netherlands (CCN)-Amsterdam UMC, Flevo Hospital, Haaglanden Medical Centre (HMC), Isala Hospital, Máxima Medical Centre (MC), Meander MC, Northwest Clinics (NWZ) Alkmaar, Onze Lieve Vrouw Gasthuis (OLVG) West, Saxenburgh Medical Centre (SXB) Hardenberg, Wilhelmina Hospital Assen (WHA) and Hospital Gelderse Vallei (ZGV). Amsterdam UMC, Máxima MC, Meander MC and OLVG West collaborate with Dutch Olympic Committee*Dutch Sports Federation (NOC*NSF), as cardiology High-Performance Partners of TeamNL. Research projects approved by institutional review board (IRB) are carried out at Amsterdam UMC and Máxima MC. *ADRZ* Admiraal de Ruyter Hospital, *AMC* Amsterdam Medical Centre, *CWZ* Canisius-Wilhelmina Hospital, *MST* Medical Spectrum Twente, *RKZ* Red Cross Hospital, *SKB* Regional Hospital Queen Beatrix, *VUmc* Free University medical centre

In total, 46 centres (52% of NVVC-registered cardiology departments or clinics) responded to the survey (1 centre located in Belgium). Twenty-eight centres (61%) reported that they had a specific outpatient clinic for sports cardiology or had one or more cardiologists with expertise in sports cardiology (Fig. 1). The majority (79%, *n* = 22/28) reported sports specific expertise, predominantly in soccer (71%), endurance sports/long distance running (32%), cycling (27%), speed skating (18%) or diving (18%). Collaborations with clubs or sports federations were reported by 79% of the centres (*n* = 22/28) and 14% (*n* = 4/28) reported a cardiology collaboration with NOC*NSF as so-called ‘High-Performance Partner’ of TeamNL; TeamNL comprises athletes who have been selected to represent the Netherlands internationally. Integrated multidisciplinary meetings on a regular basis were reported by 43% (*n* = 12/28), mostly attended by cardiologists and sports physicians only (58%) and less frequently by pulmonologists, radiologists or rehabilitation physicians. A small number of collaborating centres (*n* = 5/12) reported attending extensive meetings organised by the Amsterdam University Medical Centre (UMC), including participation of additional disciplines (genetics, molecular cardiology, paediatric cardiology, electrophysiology, cardiac imaging, nuclear medicine). Only two centres (Máxima Medical Centre and Amsterdam UMC) reported ongoing research projects that had been approved by an IRB (Tab. 1).

**Table 1:** Synopsis of The Netherlands Sports Cardiology Map

Cardiology department/clinic	HighPerformance Partner TeamNL	City	Primary contact (cardiologist)	Specific expertise	Collaboration with sports club or sports federation	Multidisciplinary meeting	IRBapproved research projects
*Admiraal de Ruyter Hospital*	–	Goes; Vlissingen; Zierikzee	M.H.H. de Vaan, MD, PhD	Diving	–	–	–
*Amsterdam UMC*	Yes	Amsterdam (AMC and VUmc); Papendal	H.T. Jorstad, MD, PhD	Elite sports; soccer; tennis; cycling; rowing; endurance sports; field hockey; water polo	AFC Ajax; KNLTB; KNVW; NOC*NSF	Cardiologists, sports physicians, cardiac EPs, genetics, radiologists, pulmonologist, paediatric cardiologists	Yes
*CanisiusWilhelmina Hospital*	–	Nijmegen	E.S. Zegers, MD, PhD	Soccer	N.E.C.	–	–
*CardioExpert*	–	Bunnik	R. Rienks, MD, PhD	Diving; elite sports; soccer; high altitude sports; flying; extreme sports	KNVB	–	–
*CCN, Amsterdam UMC, location AMC*	–	Amsterdam	M. Speleman, MD, PhD	–	–	At Amsterdam UMC	–
*Erasmus Medical Centre*	–	Rotterdam	T.W. Galema, MD, PhD	Soccer	Feyenoord	–	–
*Flevo Hospital*	–	Almere	N. Bijsterveld, MD, PhD	–	–	At Amsterdam UMC	–
*Haaglanden Medical Centre*	–	Leidschendam (Antoniushove) Den Haag (Westeinde)	B.J. Sorgdrager, MD, PhD	Soccer; cycling; endurances sports; field hockey; diving	ADO; KNVB	Cardiologists, sports physicians, pulmonologists	–
*Hartdokters*	–	Amsterdam KG/W; Leiden; Noordwijk	J.J. Regieli, MD, PhD	Diving; altitude sports -cycling; running	–	–	–
*Heilig Hart Hospital* *(Sportgeneeskunde.nl)*	–	Mol, Belgium; Oss	J.P.M. van Asseldonk, MD, PhD	Soccer	TOP Oss	–	–
*Hospital Gelderse Vallei*	–	Ede	M.J. van der Veen, MD, PhD	–	Sports Valley	At Amsterdam UMC	–
*Isala Hospital*	–	Zwolle	J.R. Timmer, MD, PhD	Speed skating	PEC Zwolle; Lotto Jumbo	Cardiologists and sports physicians	–
*Laurentius Hospital Roermond*	–	Roermond	R. van der Borgh, MD	Endurance sports; altitude sports	Sunweb cycling team	–	–
*Maastricht UMC*	–	Maastricht	C. Knackstedt, MD, PhD	–	Boels-Dolmans Cycling	–	–
*Martini Hospital Groningen*	–	Groningen	J.L. Posma, MD, PhD	Soccer; basketball	FC Groningen; Donar basketball	–	–
*Máxima Medical Centre*	Yes	Veldhoven	J. Hoogsteen, MD, PhD	Elite sports; speed skating; cycling; soccer	PSV; Jumbo-Visma; CSC; NOC*NSF	Cardiologists and sports physicians	Yes
*Meander Medical Centre*	Yes	Amersfoort	P.J. Senden, MD, PhD	Elite sports; soccer; cycling	FC Utrecht; NOC*NSF	Cardiologists, sports physicians, pulmonologists, radiologists	–
*Medical Spectrum Twente*	–	Enschede	M.F. Scholten, MD, PhD	–	–	–	–
*Noordwest Clinics*	–	Alkmaar	G.P. Kimman, MD, PhD	Soccer	AZ	Cardiologists and sports physicians	**–**
*OLVG West*	Yes	Amsterdam	A.R. Willems, MD, PhD	Elite sports	NOC*NSF	At Amsterdam UMC	–
*Regional Hospital Queen Beatrix*	–	Winterswijk	P. Sijbring, MD, PhD	Soccer	De Graafschap	–	–
*RKZ Beverwijk/CCN*	–	Beverwijk; IJmond	M.A.C. Koole, MD	Cycling; speed skating; running; rowing; strength training	Telstar	–	–
*St. Anna Hospital*	–	Geldrop	J.H.P. Janssen, MD, PhD	Swimming; soccer	PSV; VVV; PSV swimming; Helmond Sport	–	–
*St. Elisabeth Hospital*	–	Tilburg	A.C.B. Pronk, MD, PhD	Soccer	Willem II	–	–
*SXB Hardenberg*	–	Hardenberg	J.T. Drost, MD, PhD	Endurance sports	PEC Zwolle Woman	Cardiologists, sports physicians, rehabilitation physicians	–
*Tjongerschans Hospital*	–	Heerenveen	S.T. de Vries, MD, PhD	Speed skating; marathon; triathlon; soccer	SC Heerenveen	–	–
*UMC Utrecht, Central Military Hospital*	–	Utrecht	Prof. P.A. F. Doevedans, MD, PhD	Soccer	FC Utrecht	–	–
*Wilhelmina Hospital Assen*	–	Assen	J.K. Jongman, MD	–	–	Cardiologists and sports physicians	–

*IRB* institutional review board, *UMC* University Medical Centre, *AMC* Amsterdam Medical Centre, *VUmc* Free University medical centre, NOC*NSF Dutch Olympic Committee*Dutch Sports Federation, *cardiac EPs* cardiac electrophysiologists, *CCN* Cardiology Centre the Netherlands, *KG/W* Klein Gooioord/West, *OLVG* Onze Lieve Vrouwe Gasthuis, *RKZ* Red Cross Hospital, *SXB* Saxenburgh Medical Centre

The Netherlands Sports Cardiology Map, including the expertise listing, represents an important step towards a sports cardiology infrastructure and network medicine. According to this first survey, about a quarter of the NVVC-registered departments or clinics have expertise in sports cardiology. This suggests a high level of interest in a relatively young and developing subspecialty of cardiology. Furthermore, predominantly sports with a low-static/high-dynamic component[10] are fields of expertise; soccer was reported by the majority (71%). This potentially reflects the demand for such expertise from clubs and sports organisations and a relatively large number of departments and clinics with interest in providing such expertise. However, numerous popular sports are currently not well represented in the expertise listing, such as weightlifting, skiing, motor sports and boxing.

This first sports cardiology survey in the Netherlands was designed to make an inventory of existing initiatives, without applying any quality criteria. While a limited number of countries have established career tracks for sports cardiologists, there are initiatives aimed at addressing this matter. Sports cardiology senior residencies are currently offered in two medical centres in the Netherlands (Máxima Medical Centre and Amsterdam UMC). The NVVC and ESC are both in the process of evaluating whether fellowships in sports cardiology or preventive cardiology (with sports as an integral part) should be developed. Furthermore, the ESC offers an application process for European accreditation as an expert centre in sports cardiology. Finally, the ‘High-Performance Partner’ status, which has been offered to four cardiology departments in the Netherlands, is preceded by an internal evaluation performed by the medical staff of NOC*NSF. As the abovementioned initiatives are further developed, cardiology departments/clinics or individual cardiologists will gradually have access to different opportunities to distinguish themselves with expertise, collaborations or accreditations.

Our survey demonstrates that network medicine in sports cardiology is in development. One third of the respondent centres have already integrated multidisciplinary meetings on a regular basis, which are mostly attended by sports cardiologists and sports physicians. A number of these centres contribute to one larger multidisciplinary meeting. With the current level of interest in sports cardiology and clinical collaborations, further development of such meetings and panels seems warranted.

With only two centres reporting IRB-approved research projects, there is clearly a need for stimulation of sports cardiology research and research collaborations in the Netherlands. While this is almost certainly an underrepresentation of the real number of sports cardiology research projects, it is of concern that within the described clinical network, only a fraction of the centres are actively involved in sports cardiology research. Initiatives to integrate sports cardiology into collaborations with other disciplines, such as sports medicine, human movement science and public health, offer opportunities to advance sports cardiology research tracks. Furthermore, policy makers involved in writing knowledge agendas should urgently be made aware of the unmet needs of athletes, patients, and healthcare professionals in sports cardiology.

### Limitations

With regard to our survey and its results, a number of limitations should be taken into account. First, we performed a web-based survey based on self-reporting that was accessible to all NVVC-registered cardiologists. Therefore, all drawbacks pertaining to self-reporting apply.

Second, no quality criteria were applied to our map or listing. Future developments for accreditation or quality standards could hypothetically include levels of expertise for individuals or centres. For example, a level‑1 accreditation could entail attending a minimum of one national course (offered by the Dutch Cardiovascular Educational Institute) and one international course or congress in sports cardiology (offered by an organisation for preventive/sports cardiology, f.e. the European Association of Preventive Cardiology). In addition, a level‑2 accreditation would require a certain patient volume, additional expertise and partial accreditation according to national and European core curricula in preventive cardiology. A level‑3 accreditation could, for example, entail a full accreditation in preventive cardiology, an established multidisciplinary collaboration and active research programmes.

Third, the current sports cardiology map is designed as a static illustration; it is preferable to adopt a live platform with regular updates and links to the different centres of expertise.

Finally, our survey was not designed to collect data on educational tracks in sports cardiology. While integration of such educational tracks would be of interest to most sports cardiology professionals, this was outside the scope of the current survey.

## Conclusion

The Netherlands Sports Cardiology Map (Fig. 1) is an important step towards effective network medicine for patients, clinicians and researchers. Such a map and the accompanying listing of expertise assist individuals in easily identifying current care pathways, finding collaborators to improve educational tracks and establishing research collaborations.
